# Sustainable synthesis of graphene-based adsorbent using date syrup

**DOI:** 10.1038/s41598-019-54597-x

**Published:** 2019-12-02

**Authors:** Shaihroz Khan, Anjali Achazhiyath Edathil, Fawzi Banat

**Affiliations:** Department of Chemical Engineering, Khalifa University - SAN Campus, PO Box: 127788, Abu Dhabi, United Arab Emirates

**Keywords:** Chemical engineering, Synthesis of graphene

## Abstract

Here we demonstrate, a facile *in-situ* strategy for the synthesis of environmentally benign and scalable graphene sand hybrid using date syrup as a sustainable carbon source through pyrolysis at 750 °C. Raman and SEM images revealed that the as-prepared date syrup-based graphene sand hybrid (D-GSH) had imperfections with macroporous 2-D graphene sheet-like structures stacked on the inorganic sand support. The applicability of the D-GSH for decontaminating the water from cationic (Methyl Violet, MV) and anionic (Congo Red, CR) dye and heavy metals (Pb^2+^ and Cd^2+^) was tested. Batch experiments demonstrated that D-GSH showcased exceptional capability for both dye and heavy metals removal with fast adsorption following pseudo-second-order kinetics. The adsorption capacities for MV, Pb^2+^, and Cd^2+^ were respectively 2564, 781 and 793 mg/g at 25 °C, the highest capacity graphene-based adsorbent reported in the literature to date. In addition, D-GSH also exhibited high adsorption capacity for anionic dye, CR (333 mg g^−1^) and good recyclability (3 cycles) for all the contaminants. The thermodynamic studies further confirmed that the adsorption of all contaminants was thermodynamically feasible, spontaneous and endothermic with ∆H° of 48.38, 89.10, 16.89 and 14.73 kJ/mol for MV, CR, Pb^2+^ and Cd^2+^, respectively. Thus, utilization of a simple one-step strategy to produce graphenic sand hybrid using date syrup helped in developing a cost-effective and environmentally friendly dye and heavy metal scavenger that can be used as a one-step solution for water decontamination.

## Introduction

Water is one of the world’s valuable resources, and only 1% of the global water supply is available for consumption and domestic use. With the augmented urbanization and substantial industrialization activity, enormous amounts of hazardous chemicals are discharged into the receiving waters every day. Among the emerging inorganic and organic contaminants, heavy metals and dyes are frequently found in the industrial effluents, which if untreated become a principal concern to the environment and public health as they are non-biodegradable and tend to accumulate in living organisms^1–4^. Consequently, numerous efforts are emerged to develop cost-effective and appropriate remediation materials and technologies that can regulate the amount of these toxic and persistent water pollutants to permissible levels before being discharged to the water bodies. To date, different treatment technologies such as photodegradation^5,6^, precipitation^7,8^, coagulation & flocculation^9^, membrane separation^9,10^, and ion exchange^11,12^ have been tested for removing different harmful pollutants from wastewater. These conventional methods suffer from severe drawbacks in terms of cost-effectiveness, performance efficiency, applicability for a wide range of pollutants, or reusability. Comparatively, adsorption is a relatively mature, globally acclaimed, versatile, economically feasible, and highly efficient technology for pollutant remediation^13–16^. Traditional carbon-rich adsorbents such as charcoal, soot, and biochar are widely used because of their low cost and high surface area^17,18^. However, its condensed structures consume large sorption potential via limiting internal sites and thus cannot treat and produce adequate clean water. Moreover, owing to the increase in water demand coupled with stringent environmental guidelines to create public health sustainability, there is still an increasing demand for the development of effective carbon-based sorbents with higher surface areas, partition coefficients, and sorption capacities^19–21^.

With the advent of nanotechnology, researchers have intensely explored the use of carbon nanomaterials for water purification with the hope that it may open new fruitful pathways to curb the existing water shortage. Graphene, the newest addition to carbon family is a novel 2 D one atom thick nanomaterial sheet made of sp^2^ hybridized carbon atoms arranged in a honeycomb structure and has attracted tremendous research interests owing to its unique physicochemical and mechanical properties^22^. The high theoretical surface area (~2600 m^2^ g^−1^) combined with its versatile chemistry like electron-rich π-system and the highly hydrophobic surface has led to the use of graphene as a potential revolutionary adsorbent for environmental pollutant management^13–25^. Compared to CVD graphene, reduced graphenes are widely exploited for chemical and environmental application^26,27^. The highly hydrophobic carbon conjunction surface of graphene acts as potential adsorption sites for organic pollutants through π-π interactions. Furthermore, the presence of defects, step-edges and groove/wrinkle areas on the graphene are considered as high-surface-energy adsorption sites which are preferentially occupied by external molecules^28^. Nevertheless, a key barrier in the practicability of pristine graphene nanosheets for water purification is its high cost and post-treatment-handling, including recovery after the decontamination process as they aggregate heavily in water due to their large-area π-π interactions and strong van der Waals forces between the graphene layers^29^. To overcome these engineering issues, it is indispensable to anchor these graphene nanosheets onto an economical and reliable inorganic substrate such as sand. The preparation of these graphene sand hybrids would not only allow the full expression of the graphene adsorption sites but will also ensure dispersibility and easy separation from water. Recently, several synthetic routes have been reported for the preparation of graphene sand hybrids from diverse sources of carbon such as sugar^30,31^, gelatin^32^, palm sugar, asphalt^33^, etc. This is crucial as chemically different carbon sources are available in different parts of the world where their molecular and compositional chemistry are expected to play a key role in controlling the properties of the as-prepared graphene sand hybrids. Nevertheless, it is of paramount importance to apply an environmentally friendly and renewable raw material source for the sustainable production of graphene-based nanomaterials to cope up with the alarming challenges facing its mass production.

In this work, we proposed a single-step strategy to develop efficient and eco-friendly graphene sand hybrids using date syrup, a widely available and sustainable carbon source in the Mediterranean region via pyrolysis. Through this *in-situ* synthesis strategy, a large scale of graphenic material could be synthesized economically and immobilized on abundant desert sand without the use of any external chemical agents. It is believed that during pyrolysis the naturally abundant sucrose and fructose molecules in the date syrup undergoes complete exfoliation to form graphene nanosheets on the desert sand surface, thereby exposing the powerful adsorption sites concealed in the stacked graphene. Furthermore, an extensive investigation was carried out to understand the applicability of the as-synthesized carbonaceous material for purifying water from anionic and cationic contaminants such as dyes and heavy metals. The main novelty of the work is the development of graphene nanosheets on a desert sand surface using date syrup as a sustainable carbon source to be used as an eco-friendly graphenic adsorbent for the complete removal of heavy metals and toxic dyes from water treatment. In short, the work was designed to develop a simple, low cost, green chemistry-based graphenic adsorbent which exhibits superior separation capacity and regeneration capability for a variety of organic and heavy metals based contaminants that are ubiquitous in water. The adsorption capacity of the as-prepared adsorbent far surpassed the adsorption capacity of similar reported graphene-based adsorbents. This will undoubtedly open new avenues for the practicability of graphenic material to curb the existing water shortage.

## Experimental Section

### Synthesis of date syrup-based graphene sand hybrid (D-GSH)

**Figure 1:**
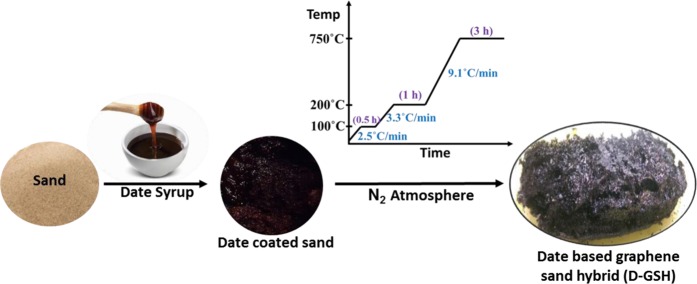
Synthesis of date syrup-based graphene sand hybrid (D-GSH).

The schematic preparation procedure of D-GSH from date syrup and desert sand is shown in Fig. 1. In general, D-GSH was produced by the graphitization of date syrup in the presence of desert sand particles, both of which are abundantly available in the United Arab Emirates (UAE). Briefly, the desert sand particles of known particle size were mixed with date syrup in the ratio 2:5 and the mixture was stirred for 1 hour, followed by overnight drying at 80 °C to evaporate excess moisture. The dried and homogeneously mixed solid product was then loaded on a ceramic boat and placed into a tube furnace for carbonization under N_2_-flow (100 mL/min.) Prior to starting the heating program, the air inside the furnace was replaced with high purity N_2_ gas. The mixture was heated to 750 °C by following the heating protocol as described in Scheme 1 to ensure complete graphitization of the date syrup. After the completion of the experiment, the furnace was allowed to naturally cool down to room temperature and the obtained carbonaceous and fluffy graphene-like material referred as “D-GSH” was further characterized and used for batch adsorption experiments.

### Batch adsorption experiments

A 1000 mg/L stock solution of dyes and heavy metals was prepared by dissolving an appropriate quantity of respective dyes or heavy metal salts in deionized water, and solutions of desired concentrations were prepared by successive dilution of the stock solution. Batch mode of adsorption experiments was performed in 25 mL conical flasks containing 10 mL of contaminant solution with a known initial concentration. A fixed quantity of D-GSH adsorbent was added and the stoppered conical flasks containing the mixtures were shaken mechanically in a temperature-regulated water bath (Dihan, Korea) at a constant speed of 140 rpm and predetermined temperature and time. Detailed methods regarding the effect of various experimental conditions such as sand particle size, adsorbent dosage, solution volume, pH, time, initial concentration, and temperature and data analysis of sorption kinetics and isotherms of dyes and heavy metals were given in the Supporting Information. On completion of adsorption experiments, supernatant samples were withdrawn, and the residual dye concentration was analyzed using UV-vis spectrophotometer (Brookhaven Instruments Corp., Holtsville, NY, USA) at the maximum wavelengths of 590 nm for MV, 497 nm for CR, 668 nm for MB and 507 nm for MO. The residual heavy metals concentration was analyzed using an inductively coupled plasma optical emission spectrophotometer (ICP-OES, Perkin Elmer). All the experiments were performed in duplicates, and the reported results are average values ± standard deviation. The percentage removal and amount of dye/heavy metal contaminant adsorbed at equilibrium (*q*_*e*_), was calculated using the Eqs. (1) and (2), respectively.

1 $${\rm{Removal}}( \% )=\frac{{C}_{0}-{C}_{e}}{{C}_{0}}\ast 100$$

2 $${q}_{e}=\frac{V}{m}({C}_{0}-{C}_{e})$$

Where, *C*_0_ and *C*_*e*_ are the initial and equilibrium concentrations of dye/heavy metal contaminant (mg/L), respectively, m is the dry mass of D-GSH (g), V is the volume of the contaminant solution (L).

Furthermore, the reusability and regeneration of the adsorbent and selectivity towards heavy metals and organic dyes were investigated and the details of which are provided in the Supporting Information.

## Results and Discussion

### Adsorbent synthesis and characterization

**Figure 2:**
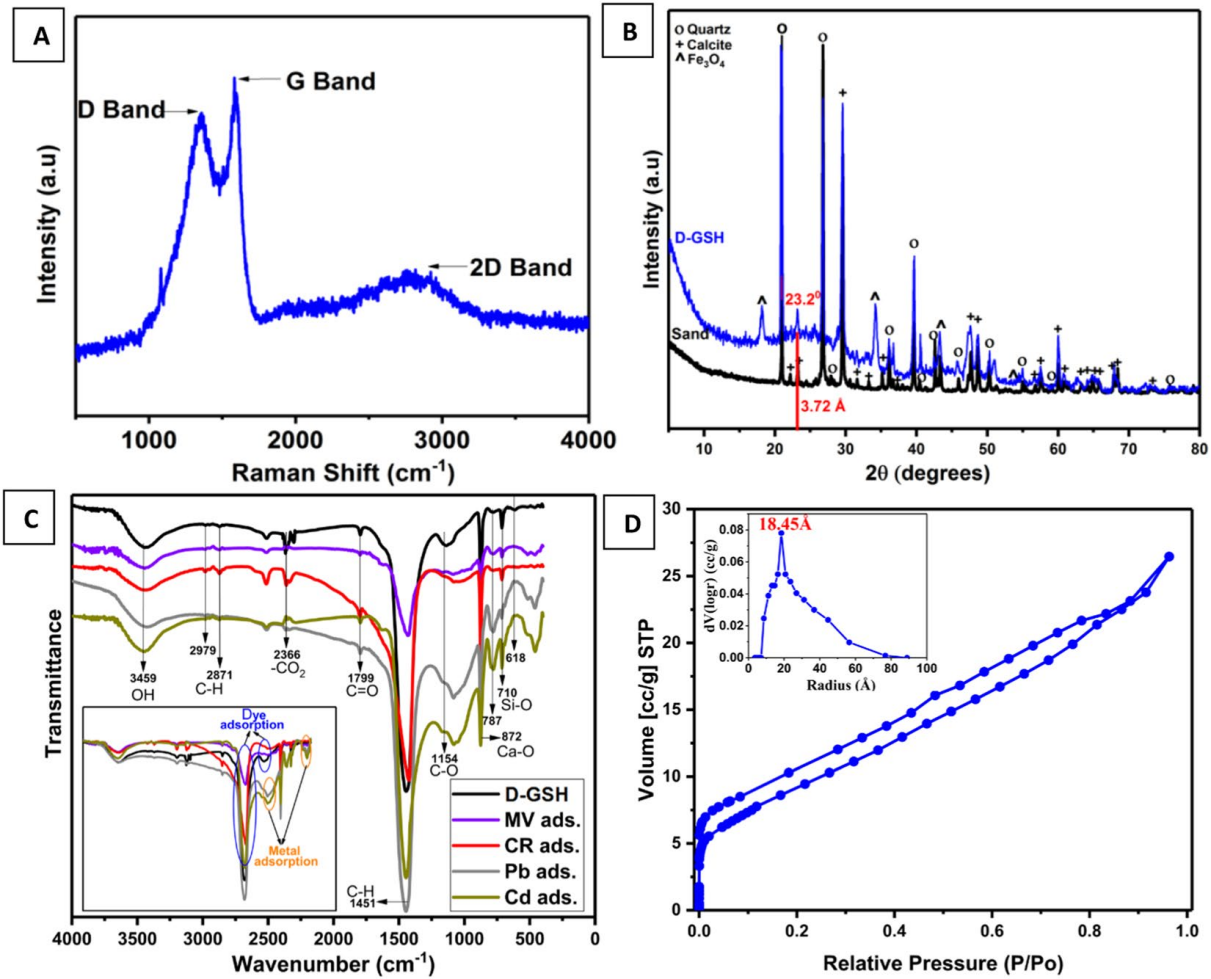
(**A**) Raman Spectroscopy of D-GSH, (**B**) XRD of sand and D-GSH, (**C**) FTIR analysis of fresh, MV, CR, Pb and Cd adsorbed D-GSH samples and (**D**) Nitrogen adsorption-desorption isotherm and BJH pore size distribution (inset) of D-GSH.

Figure 1 illustrates the novel green method used for the preparation of graphenic organic-inorganic hybrid material using date syrup as the biomass source. In this *in-situ* synthesis method, firstly date syrup was immobilized on sand without the use of any binder by drying at 80 °C. Upon further heating at 200 °C in an inert atmosphere, the syrup was converted to graphene-based carbon through carbonization, followed by complete graphitization at 750 °C. This results in the formation of date based graphenic sand hybrid material (D-GSH) with a strongly adhered char deposit on the surface. The Raman spectrum shown in Fig. 2A clearly depicts three distinct peaks at 1370 cm^−1^, 1580 cm^−1^, and 2760 cm^−1^, corresponding to D (defects and disorder), G (graphitic) and 2D bands (number of graphene layers), respectively. These results reveal that D-GSH has largely *sp*^2^ frame graphene-like carbon material obtained by the conversion of sucrose and fructose molecules present in the date syrup. The intensity ratio, I_D_/I_G_ of 0.94 indicates that D-GSH has a structural disorder same as that of graphene oxide^34^. On the other hand, the I_2D_/I_G_ ratio was found to be 0.43, attributed to the presence of multilayer graphene on D-GSH^35^. The powdered X-ray diffraction (XRD) spectra of D-GSH showed (002) a sharp peak at ~23.2° (2θ) with an interlayer spacing of 3.72 Å and could be attributed to the (002) diffraction pattern of multilayered graphenic structures present on the surface of the D-GSH. The spectra also depicted several other small and medium peaks at various 2θ values and these could be assigned to the highly crystalline desert sand support used in this study and corresponds to the characteristic diffraction pattern of quartz and silica as indicated by the symbols ‘o’ and ‘ + ’, respectively in Fig. 2B. As the graphene sheets are spread over the sand particles, the diffraction patterns of D-GSH are observed to be significantly lower than that of sand support^36^. Furthermore, the XRD spectrum of D-GSH showed the presence of few additional peaks at 2θ of 18° and 34.2° corresponding to the (111) and (311) plane of Fe_3_O_4_ particles, respectively^37^. These peaks confirm the presence of iron particles present in the date syrup. Both Raman and XRD indicate that the as-prepared D-GSH is in close relation to the graphenic material reported in literature^30,31,33,38^.

The porosity of D-GSH was characterized using the nitrogen adsorption/desorption isotherms at 77 K and pore-size distribution plot of D-GSH calculated using the Barrett–Joyner–Halenda (BJH) method (Fig. 2C). The isotherm curve followed type II isotherm with H4 type hysteresis loop, suggesting that D-GSH has typical mesoporous structures with an average BJH pore radius of 18.45 Å and cumulative pore volume of 0.041 cc/g. The specific surface area calculated using the Brunauer−Emmett−Teller (BET) model was found to be 22.4 m^2^/g. Even though pristine graphene sheets have a high theoretical surface area (2630 m^2^/g), D-GSH exhibited a relatively low surface area. The surface area of D-GSH largely depends on the sand particles present in the hybrid material when graphene was coated on the sand. This is in agreement with the results reported by Yang *et al*.^29^, wherein the specific surface area of pristine graphene sheets reduced drastically when it was coated onto SiO_2_ particles.

The presence of graphene on sand particles as well as the interaction of the functional groups of D-GSH in the adsorption of organic dyes and heavy metals were demonstrated using Fourier transform infrared (FTIR) spectroscopy analysis (Fig. 2D). In the FTIR spectrum of fresh D-GSH, strong peaks at 1796 and 1451 cm^−1^ attributed to C=O stretching of –COOH groups and C-H stretching vibrations, respectively; were present. These observations serve as a piece of evidence for the complete graphitization of sucrose and fructose fragments on sand surface^36^ resulting in a carbonaceous adsorbent with many oxygen functional groups (i.e.–OH, –COOH, etc.) which can act as binding sites for contaminants. Furthermore, strong absorption bands at 792 and 707 cm^−1^ consistent with the symmetric Si-O stretching and bending vibrations of the SiO_2_ functional group were conspicuously present in the spectra, which confirms the presence of quartz in the sand^39^. Whereas, the sharp band observed at 874 cm^−1^ can be assigned to the Ca-O stretching of the calcium carbonate species present in the sand^40^. On the other hand, after dye adsorption, the band intensity at 1451 cm^−1^ was reduced significantly and shifted to a lower wavelength of 1420 cm^−1^ and the peak at 1154 cm^−1^ completely disappeared owing to the utilization of functional groups during adsorption. A new band was formed at 1172 cm^−1^ indicating the presence C-N stretching after dye adsorption. After heavy metal adsorption, a new absorption band at 1154 cm^−1^ with remarkable intensity and vibrational bands attributed to metal oxide (Cd-O and Pb-O) appeared at low wavenumbers (518 and 457 cm^−1^) of the spectrum, further verifying the successful interaction of metal ions onto the binding sites of D-GSH adsorbents through complexation^41^.

**Figure 3:**
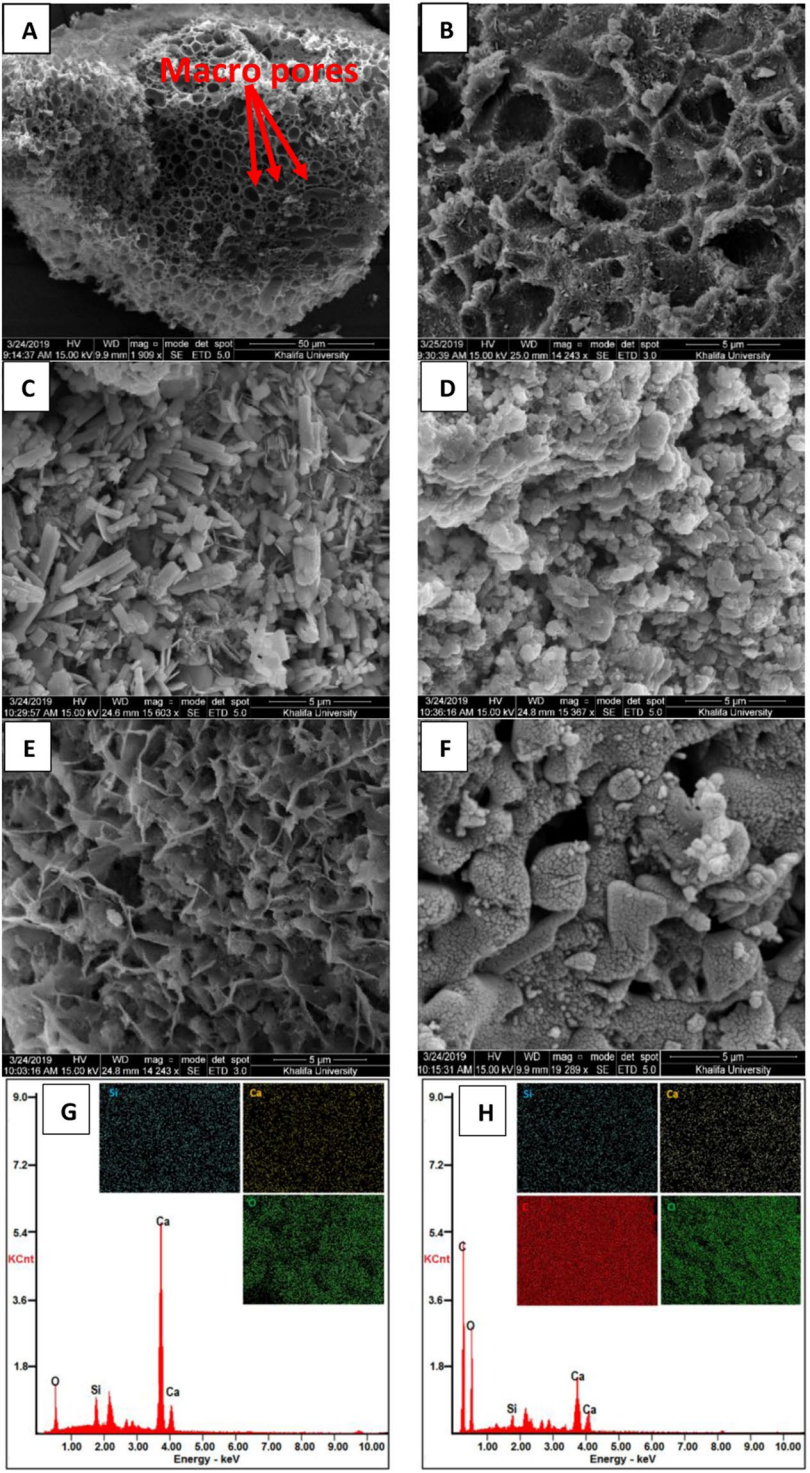
SEM images of (**A**,**B**) fresh D-GSH at low and high magnification, (**C**) MV, (**D**) CR, (**E**) Pb and (**F**) Cd adsorbed D-GSH samples and the EDX spectrum of (**G**) sand and (**H**) fresh D-GSH samples. The inset of (**G,H**) shows the corresponding elemental maps (silica colored in cyan, calcium colored in yellow, oxygen colored in dark green and carbon colored in red).

The microscopy observations depicted in Fig. 3 revealed the morphology and structure of the as-synthesized D-GSH hybrids, prepared by coating date syrup on the sand and after dye and heavy metal adsorption. From Fig. 3A,B, it can be seen that fresh D-GSH had honeycomb-like-graphenic morphology owing to the hybridization of the sand surface with an interlinked network of multilayered graphene sheets^42^. Along with the layered structure, the extremely rough and wrinkled surface with predominantly macropores helped in maintaining a high active surface. Theoretically, these characteristics are believed to play a crucial role in providing active sites for dye and heavy metal adsorption. Furthermore, it was noteworthy to mention the presence and uniform distribution of C element on the corresponding EDX spectrum of D-GSH (Fig. 3G,H) compared to pristine sand, confirming the successful hybridization of graphene on the sand. Conversely, after adsorption (Fig. 3C–F), SEM images indicated a drastic change in morphology and the surface was uneven owing to the presence of agglomerated contaminant particles adsorbed all over the sand surface. The corresponding EDX spectrum along with elemental mapping of adsorbed samples (Please refer Figure S1 in the Supplementary Information) confirmed the presence and uniform distribution of N, N and S, Cd and Pb elements further, indicating that MV, CR, Pb, and Cd are successfully loaded onto the D-GSH surface forming a uniform network structure.

To further clarify the microstructure of the D-GSH and the distribution of graphene over sand, HR-TEM investigations were conducted. D-GSH has a relatively larger particle size owing to the wrapping of graphenic sheets over the sand particles of size ranging from 200–500 µm. In order to observe the nature of graphenic nanosheets using TEM, D-GSH samples were sonicated in ethanol for 2 hours and few drops of the supernatant solution were transferred to 400 mesh formvar copper electron microscopy grids (coated with holey carbon film). The so-obtained HR-TEM images (Figure S2 in the Supplementary Information) further unveiled that the D-GSH has an interlinked network of multilayered graphene sheets covering the sand particles, which is in good agreement with the SEM results.

### Dye adsorption experiments

**Figure 4:**
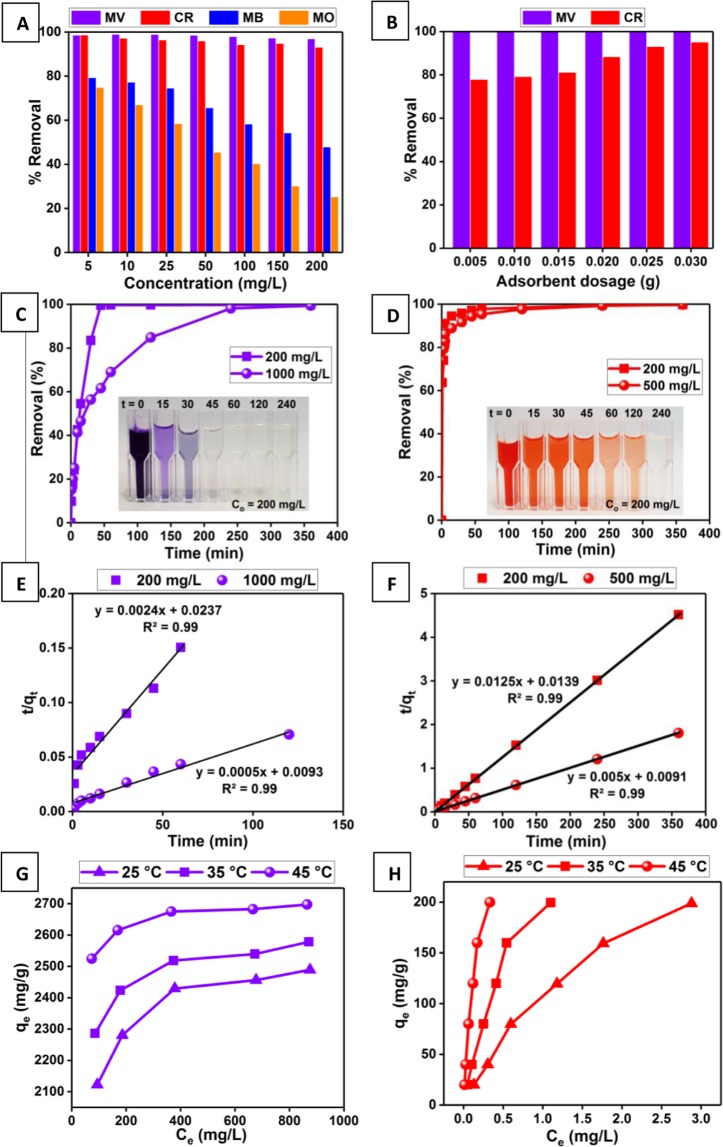
(**A**) Preliminary dye adsorption studies, (**B**) Effect of adsorbent dosage on MV and CR adsorption, (**C,D**) Effect of contact time on MV and CR adsorption, respectively, (**E,F**) Plots of pseudo-second-order kinetic models for MV and CR, respectively. [Straight lines are the fitting results], (**G,H**) Effect of initial concentration and temperature on MV and CR dye uptake capacity, respectively.

The development of an adsorbent material that can remove both anionic and cationic dyes has promising application prospects especially for treating colored wastewater from the industry. To asses the effectiveness of the prepared adsorbents in removing organic dyes from wastewater, preliminary adsorption experiments were carried out using the as-prepared D-GSH and desert sand as adsorbents. Firstly, a series of dye solutions such as MV & MB (cationic) and CR & MO (anionic) with varying initial concentrations in the range of 5 to 200 mg/L were exposed to the D-GSH and desert sand (DS) (25.0 mg) for 4 hours to reach an equilibrium. From the results (Fig. 4A), it is evident that the DS had a negligible dye removal capacity. On the other hand, the D-GSH adsorbent material showed fairly good adsorption of MB and MO dye up to an initial concentration of 25 mg/L (~75%) and 10 mg/L (~67%), respectively, while further increase in concentration drastically decreased the removal efficiency. However, for MV and CR dyes, the % removal was almost constant with >99% for MV and >93% for CR at an initial dye concentration of 200 mg/L, while bare sand removed only a negligible amount of dyes. Thus, the preliminary results confirmed that the conversion of sugar from date syrup to graphitic carbon on the sand surface had significantly enhanced the dye adsorption capacity of desert sand. As the highest removal efficiency was showcased for MV and CR, subsequent adsorption studies were carried out using these dyes as model pollutants to demonstrate the potential of D-GSH as an economical adsorbent for colored wastewater treatment. Furthermore, the indigenous availability of dates in the Mediterranean region makes the one-pot synthesis of the graphene-based adsorbent using date syrup an environmentally benign and scalable process with no chemical footprint.

Firstly to optimize the parameters affecting the adsorption, the effect of sand particle size on dye removal performance was evaluated. It was found that MV dye removal efficiency remained almost constant (>99%) with an increase in sand particle size, whereas CR dye removal efficiency slightly decreased from 96% to 86%. This conclusion was drawn after performing batch MV and CR adsorption experiments using the as-synthesized D-GSH adsorbent on desert sand supports of various particle sizes such as 0 to 200, 200 to 500, 500 to 1000 and 1000 to 1500 µm (Figure S3–A in the Supplementary Information). Even though smaller sand particles gave slightly higher CR removal capacity owing to the larger surface area available for adsorption, separation becomes a difficulty when particles get finer and smaller. Thus, subsequent dye adsorption experiments were carried using D-GSH with sand particle size between 200–500 µm in order to ensure high removal efficiency as well as easy separation. Figure 4B shows the MB and CR dye adsorption capacity when different amounts of D-GSH (5.0 to 30.0 mg) were used. It was seen that, for CR adsorption, the removal % increased with the addition of adsorbent. This is because as the adsorbent dosage increased, more adsorption sites became available leading to an increase in the removal efficiency^43,44^. The highest removal efficiency of 93% was obtained at a dose of 25.0 mg owing to the increase in the availability of the active adsorption sites. Further increase in dosage did not show any improvement in removal efficiency due to the agglomeration of the adsorbent particles leading to longer diffusive path lengths for dye molecules^45,46^. Moreover, on increasing the D-GSH dosage beyond 25.0 mg, the CR adsorption capacity was reduced. Thus, an optimal adsorbent dosage of 25.0 mg/10.0 mL was chosen for all subsequent CR dye adsorption experiments. On the other hand, increasing the D-GSH dosage showed a negligible effect on MV adsorption. For a fixed volume of dye solution (10 mL), with the decrease in W/V ratio, the MV uptake capacity of D-GSH increased from 399.8 mg/g to 1983.9 mg/g, while 99.9% MV removal was attained even when the initial MV concentration was 1000 mg/L. This is because 5.0 mg of D-GSH was enough to adsorb the dye contaminants present in 10 mL of solution providing more uptake per gram of the adsorbent. The decrease in uptake capacity with an increase in adsorbent amount was attributed to the presence of some unsaturated sites available on D-GSH during adsorption^47^. For the same initial MV concentration, varying the MV dye solution volume from 10 to 20 mL for a fixed weight of D-GSH, say 5.0 mg (Figure S3–B in the Supplementary Information), the uptake capacity increased from 1983.9 to 2277.2 mg/g. With the increase in solution volume (at lowest W/V), the amount of MV in solution was increased causing more amount of dye to be adsorbed to reach the adsorption-desorption equilibrium. Thus, optimum dosage to solution volume obtained by using 20 ml of MV solution volume and 5.0 mg of D-GSH adsorbent was selected for further MV dye adsorption.

The effect of the kinetics of MV and CR uptake was tested by measuring the adsorption efficiency at different time intervals and the data are as shown in Fig. 4C,D, respectively. From the results, it is apparent that the adsorption of MV by D-GSH was initially slow and gradually reached equilibrium within 45 minutes (99% removal) and 4 hours (98% removal) for 200 and 1000 mg/L MV concentration, respectively. Conversely, the kinetics of CR uptake was rapid in the beginning by removing 94% within 15 and 45 minutes for 200 and 500 mg/L CR concentration, respectively and gradually decreased as time progress and finally attained equilibrium in 3 hours as evident from Fig. 4D. The adsorption capacity of D-GSH improved drastically upon increasing the *C*_0_value of MV from 200 mg/L (*Q*_*e*_ =  399.15 mg/g) to 1000 mg/L (1987.98 mg/g). This increase is attributed to the increase in the number of available MV molecules which provides a higher driving force for overcoming the diffusion resistance between the adsorbate and adsorbent phases. A similar trend was observed during CR adsorption as well. The experimental MV and CR adsorption kinetic data were quantitatively modeled using pseudo-first order^48^ and pseudo-second order^49^ models. The mathematical representations of these models, as well as the calculated kinetic parameters obtained for both MV and CR adsorption, are given in the Supplementary Information (Table S1). The analysis showed that the experimental kinetic data for MV and CR adsorption fitted very well with the pseudo-second-order model with a correlation coefficient of *R*^2^ > 0.99 at all concentrations (Fig. 4E,F) than the pseudo-first-order model (Figure S3–C,D in the Supplementary Information). Thus, during the adsorption of MV and CR, chemisorption is likely followed. Furthermore, the calculated *Q*_*e*_ values using pseudo-second-order models showed a good agreement with the experimental *Q*_*e*_ values obtained during MV and CR adsorption.

As D-GSH exhibits extremely high adsorption capacity for MV, to access the overall efficiency of D-GSH aqueous solution with varying MV concentrations in the range of 0 to 1000 mg/L were exposed to 1.0 mg of D-GSH adsorbent and at different temperatures. Whereas for CR, the experiments were performed by exposing using 25.0 mg of D-GSH. The adsorption equilibrium results depicted in Fig. 4G,H were quantified by fitting the experimental data to various linearized adsorption isotherm models, namely: Langmuir^50^, Freundlich^51^, and Tempkin^52^ (Table 1). The quantitative analysis showed a remarkable correlation of experimental data to the Langmuir model with a high satisfactory *R*^2^ (> 0.99) at all temperatures; suggesting that adsorption of MV and CR on D-GSH was a monolayer process wherein the active sites are distributed homogenously on the adsorbent surface^50^. As presented in Table 1, *Q*_*e*_ of D-GSH for MV was extraordinarily high (2564 mg/g) at 25 °C. This remarkable removal capacity of D-GSH compared to pristine desert sand further indicated the potential adsorption capacity of the well-dispersed graphene on the sand surface. To author’s best knowledge, D-GSH is the highest-capacity ranked adsorbent known to date for MV adsorption; five times greater than that of previously reported graphene-based adsorbents (Table S2 in the Supplementary Information). Furthermore, D-GSH also achieved a significantly high *Q*_*e*_ of 333 mg/g for CR, superior to most of the graphene-based adsorbents reported in the literature (Table S2 in the Supplementary Information). The feasibility of the adsorption process which was further confirmed from the thermodynamic parameters (Table 1) suggests that the sorption is thermodynamically feasible, spontaneous and endothermic for MV and CR. The easy availability of the carbon precursor and sand support, as well as the advantage of having extraordinary dye adsorption capacity, makes D-GSH a promising alternative to any traditional adsorbent for colored wastewater treatment.

**Table 1:** Various isotherm models and thermodynamic parameters for the adsorption of dye and heavy metal contaminants onto D-GSH.

Parameters	Type of Contaminant											
	*MV*			*CR*			*Pb*^2+^			*Cd*^2+^		
	25 °C	35 °C	45 °C	25 °C	35 °C	45 °C	25 °C	35 °C	45 °C	25 °C	35 °C	45 °C
**Langmuir Isotherm**^41^ $[\frac{{\bf{1}}}{{{\boldsymbol{q}}}_{{\boldsymbol{e}}}}=\frac{{\bf{1}}}{{{\boldsymbol{q}}}_{{\boldsymbol{\max }}}\,}+\frac{{\bf{1}}}{{{\boldsymbol{q}}}_{{\boldsymbol{\max }}}{{\boldsymbol{K}}}_{{\boldsymbol{L}}}{{\boldsymbol{C}}}_{{\boldsymbol{e}}}}]$												
***q***_***max***_ (mg g^−1^)	2564.10	2631.58	2702.70	333.33	344.83	357.14	781.25	806.45	840.34	793.65	826.45	854.70
***K***_***L***_ (L mol^−1^)	0.05	0.08	0.18	0.48	1.26	4.67	2.67	3.21	4.11	5.63	6.59	8.19
***R***^2^	0.99	0.99	0.99	0.99	0.99	0.99	0.99	0.99	0.99	0.99	0.99	0.99
**Freundlich Isotherm**^42^ $[\log \,{{\boldsymbol{q}}}_{{\boldsymbol{e}}}-\,\log \,{{\boldsymbol{K}}}_{{\boldsymbol{f}}}=\frac{{\boldsymbol{1}}}{{\boldsymbol{n}}}\,\log \,{{\boldsymbol{C}}}_{{\boldsymbol{e}}}]$												
***K***_***F***_ (mg^1−1/n^ L^1/n^g^−1^)	1566.80	1857.77	2274.03	100.90	221.27	529.01	12.72	15.09	17.70	27.60	31.15	36.68
***n***	14.29	20.34	38.49	1.32	1.31	1.38	1.41	1.43	1.45	1.73	1.76	1.77
***R***^2^	0.94	0.95	0.93	0.98	0.98	0.98	0.94	0.93	0.94	0.96	0.95	0.94
**Tempkin Isotherm**^43^ $[{q}_{e}=\frac{RT}{{b}_{T}}ln\,{A}_{T}\,{C}_{e}]$												
***b***_***T***_ (J mol^-1^)	16.36	21.41	36.49	42.14	43.11	46.31	26.28	26.12	26.59	32.17	32.16	32.35
***A***_***T***_ (L g^-1^)	6.47E + 03	2.89E + 06	2.49E + 14	7.97	21.78	83.49	0.86	1.04	1.29	2.50	3.01	3.88
***R***^2^	0.94	0.95	0.93	0.96	0.95	0.97	0.90	0.90	0.88	0.83	0.83	0.82
**Thermodynamic Study** $[\varDelta {\boldsymbol{G}}=-{\boldsymbol{RTln}}{{\boldsymbol{K}}}_{{\boldsymbol{L}}}\,]$ **and** $[\mathrm{ln}\,{{\boldsymbol{K}}}_{{\boldsymbol{L}}}=\frac{\varDelta {\boldsymbol{S}}}{{\boldsymbol{R}}}-\frac{\varDelta {\boldsymbol{H}}}{{\boldsymbol{RT}}}]$												
∆G°_Exp_ (kJ mol^−1^)	−7.55	−8.95	−11.31	−14.42	−17.36	−21.38	−2.43	−2.99	−3.73	−4.28	−4.83	−5.56
∆H° (kJ mol^−1^)	48.38			89.10			16.89			14.73		
∆S° (J mol^−1^)	187.17			346.81			64.75			63.71		
∆G°_Calc_ (kJ mol^−1^)	−7.40	−9.27	−11.14	−14.25	−17.72	−21.18	−2.40	−3.05	−3.70	−4.25	−4.89	−5.53

### Heavy metal adsorption experiments

**Figure 5:**
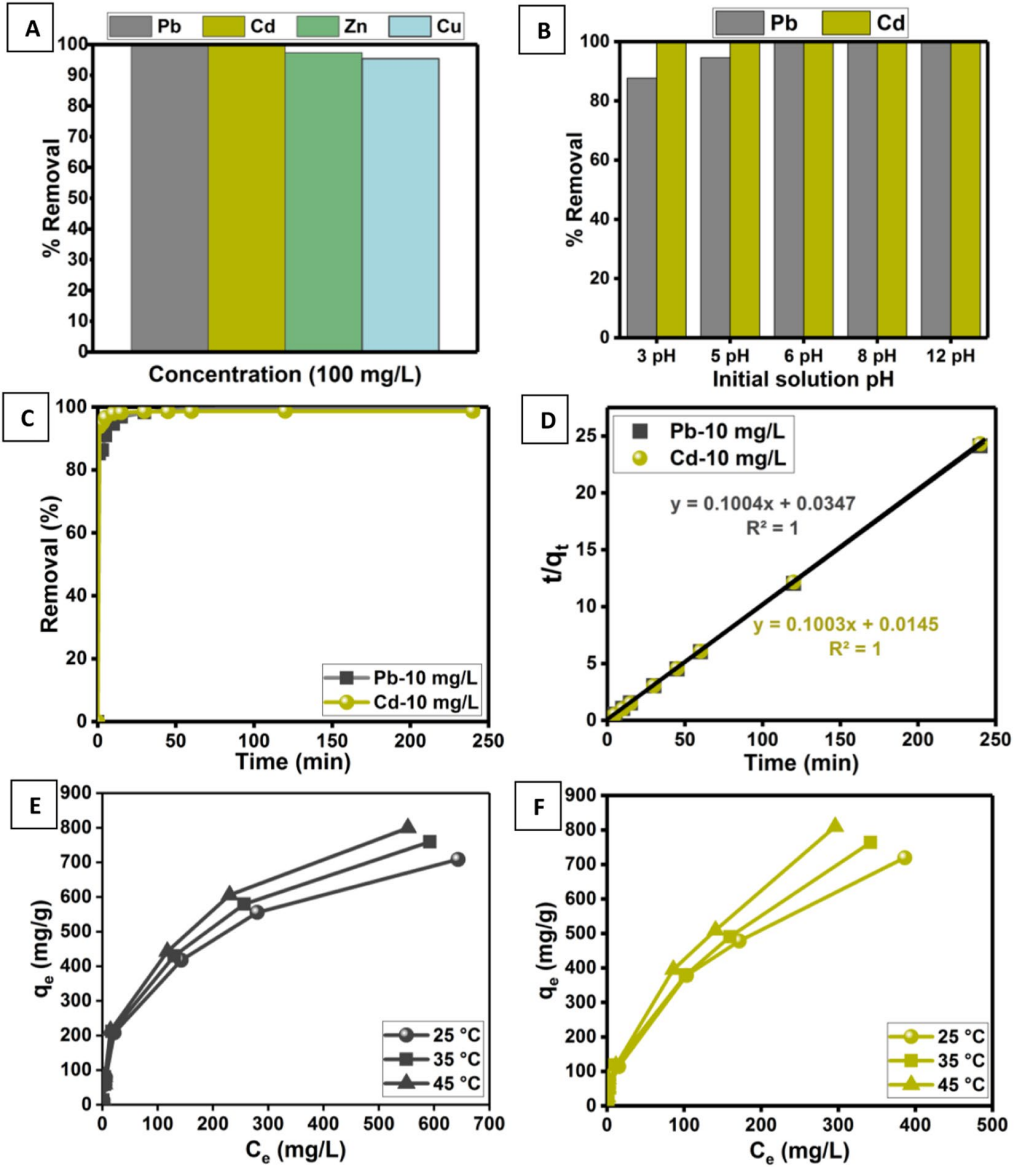
(**A**) Preliminary heavy metal adsorption studies using Pb^2+^, Cd^2+^, Zn^2+^ and Cu^2+^ ions, Effect of (**B**) initial solution pH and (**C**) contact time on adsorption of Pb^2+^ and Cd^2+^, (**D**) Plots of pseudo-second-order kinetic models for Pb^2+^ and Cd^2+^ removal [Straight lines are the fitting results] and (**G,H**) Effect of initial concentration and temperature on Pb^2+^ and Cd^2+^ uptake capacity, respectively.

The excellent dye adsorption ability encouraged us to further investigate the metal ion binding ability of D-GSH in aqueous solution. Furthermore, the removal of heavy metals is of great concern owing to its high toxicity to both human beings and ecosystems. Interestingly, the preliminary adsorption experiment (Fig. 5A) carried using various heavy metals such as Pb^2+^, Cd^2+^, Zn^2+^, Cu^2+^ revealed that D-GSH showcased higher removal efficiencies for all metal ions (99.87% for Pb^2+^, 99.62% for Cd^2+^, 97.35% for Zn^2+^ and 95.38% for Cu^2+^). Considering the slightly higher removal efficiency, Pb^2+^ and Cd^2+^ were chosen as model heavy metal pollutants for subsequent experiments. Similar to MV dye adsorption, Pb^2+^ and Cd^2+^ removal efficiency remained almost constant (>99%) for sand particle size ranging from 0 to 200 and 200 to 500 µm (Figure S4-A in the Supplementary Information). However, to ease the separation process, subsequent heavy metal adsorption experiments were carried using D-GSH with sand particle size between 200–500 µm. The dependence of initial solution pH on Pb^2+^ adsorption (Fig. 5B) increased with an increase in pH and showed the maximum at original solution pH of 6.27, while for Cd^2+^ adsorption the removal remained same irrespective of the initial solution pH. Thus, the above results indicate the removal of heavy metals is mainly rendered by the ion-chelating negatively charged functional groups present on D-GSH surface^53,54^. The effect of adsorbent dosage on Pb^2+^ and Cd^2+^ adsorption at two different concentrations (10 mg/L and 100 mg/L) are depicted in Figure S4-B,C in the Supplementary Information. It was interesting to find that, 10.0 mg of D-GSH was enough to completely remove both the heavy metals regardless of the initial metal concentration. Beyond 10.0 mg, no further improvement in removal efficiency was observed due to the agglomeration of adsorbent particles resulting in an increase in the diffusive path lengths for metal ions^45,46^.

The adsorption kinetic data presented in Fig. 5C revealed that rapid adsorption occurred during the first 5 min by removing 91% and 96% of Pb and Cd, respectively and then gradually attained an equilibrium within 45 min. Consequently, only a contact time of 45 min is required to achieve saturated adsorption of heavy metals onto D-GSH. Similar to the adsorption results for cationic dye, quantitative analysis using various kinetic models confirmed that the adsorption of Pb^2+^ and Cd^2+^ onto D-GSH also followed pseudo-second-order (Fig. 5D) with a correlation coefficient, *R*^2^ of 0.99 (Figure S4-D in the Supplementary Information). In addition, the calculated *q*_*e*_ values (Table S1 in the Supplementary Information) obtained from the pseudo-second-order model fits well with the experimentally obtained *Q*_*e*_values than pseudo-first-order, suggesting chemisorption nature of adsorption. The adsorption capacity of both Pb^2+^ and Cd^2+^ onto D-GSH increased with increasing the concentration from 0 to 1000 mg/L (Fig. 5E,F). Furthermore, the equilibrium data were modeled using Langmuir^50^, Freundlich^51^, and Tempkin^52^ isotherms to explain how the heavy metals are distributed between the phases of D-GSH absorbent and water (Table 1). The very low *R*^2^ value <0.99 at all temperatures indicates that the adsorption of Pb^2+^ and Cd^2+^ onto D-GSH does not follow the Freundlich or Tempkin model, rather follows the Langmuir model with an *R*^2^ > 0.99 (consistent with the findings obtained for cationic dyes molecules). These results essentially support that D-GSH has a homogeneous surface that can adsorb a monolayer of heavy metal ions and the removal efficiency of D-GSH would drop sharply once these available surface sites are filled-up. The monolayer Pb^2+^ and Cd^2+^ adsorption capacity, *q*_*max*_ of D-GSH was estimated to be about 781 mg/g and 793 mg/g, respectively at 25 °C. The determined adsorption capacity for Cd^2+^ and Pb^2+^ ions outperformed all other previously reported graphene-based adsorbents (Table S3 in the Supplementary Information). In addition, the performance of the D-GSH increased with the increase in temperature with a positive value of enthalpy (∆H°) and entropy (∆S°) as summarized in Table 1. From the negative value of standard Gibb’s free energy change (∆G°), it can be deduced that the thermodynamic process was spontaneous and feasible for Pb^2+^ and Cd^2+^ ions. Moreover, the increase in negative ∆G° values with increasing temperature shows an increased probability of adsorption at higher temperatures, which is consistent with the earlier reports. Overall, the high heavy metal adsorption ability of D-GSH and the ease of its production using green chemistry introduces D-GSH as an ideal adsorbent for the treatment of contaminated waters.

### Simultaneous adsorption experiments

**Figure 6:**
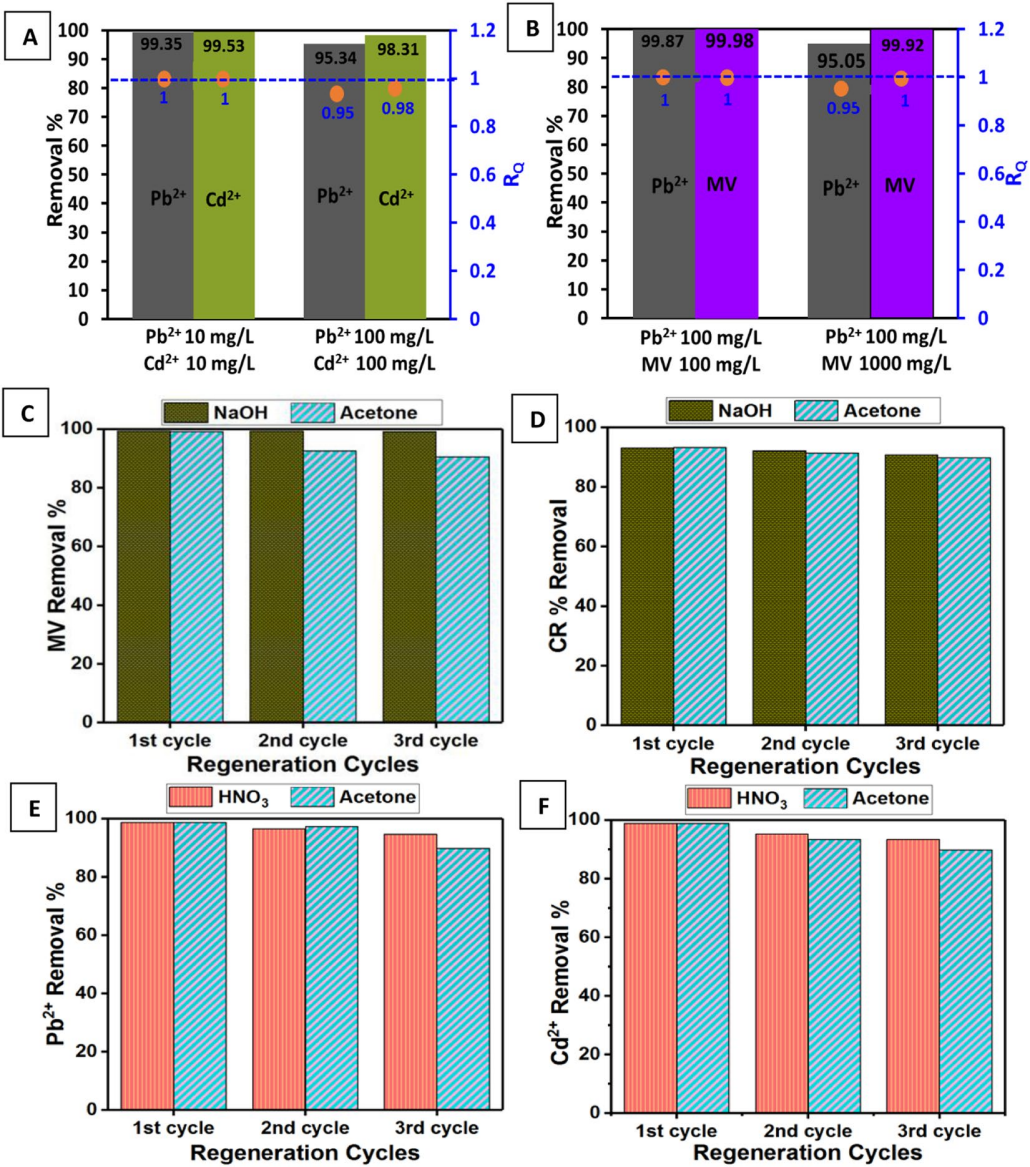
Simultaneous adsorption studies in (**A**) Pb^2+^-Cd^2+^ and (**B**) Pb^2+^-MV multi-component system, Regeneration efficiency of (**C**) MV, (**D**) CR, (**E**) Pb^2+^ and (**F**) Cd^2+^ in three consecutive cycles of desorption using various eluents.

Real wastewater contains a mixture of heavy metals as well as organic dyes. Hence, it is of utmost importance to test the selectivity of D-GSH in a multi-component system consisting of both heavy metals as well as dyes. Single solute experiments indicate that D-GSH can potentially remove more Cd^2+^ than Pb^2+^ ions. To verify this, the adsorption experiments were investigated under the same initial concentration of 10 and 100 mg/L, and the results are presented in Fig. 6A. At lower concentrations, Pb^2+^ and Cd^2+^ ions were almost completely removed from the aqueous solution using 25.0 mg of D-GSH. However, at a higher concentration of 100 mg/L, the removal efficiency of Pb^2+^ (95.34%) was slightly lower than Cd^2+^ (98.31%). On the other hand, in a solution containing a mixture of MV dye and Pb^2+^ heavy metals with each having an initial concentration of 100 mg/L, D-GSH successfully removed 99.99% of dye as well as 99.75% of Pb^2+^ ions (Fig. 6B). While at a higher concentration of MV dye solution, even though MV dye was completely removed, the adsorption of the Pb^2+^ reduced to 95.34%. Thus, in order to clearly uncover the interaction between each metal ions during simultaneous adsorption, the ratio of sorption capacities was calculated using the Eq. (1) as described below^55^:

3 $${R}_{Q}=\frac{{q}_{m,j}}{{q}_{s,j}}$$

Where, *q*_*s,j*_ (mg/g) and *q*_*m,j*_ (mg/g) are the amount of contaminant j adsorbed in a single and multi-component system with equal concentration, respectively. From the values of *R*_*Q*_ the interaction between each contaminant can be verified as follows: (i) when *R*_*Q*_ <1, antagonism, i.e., the presence of the concomitant pollutant inhibits the adsorption of contaminant j; (ii) when *R*_*Q*_ = 1, non-interaction, i.e., both contaminants do not affect each other; (iii) when *R*_*Q*_ >1, synergism, i.e., the presence of the concomitant pollutant enhances the adsorption of contaminant j. From the *R*_*q*_values obtained at the different initial concentration in the multi-component heavy metal system (Fig. 6A), we can infer that at low concentration the adsorption of heavy metals is almost unchanged (${R}_{q,P{b}^{2+}}$ and ${R}_{q,C{d}^{2+}}$ = 1). Interestingly, the sorption capacity of each heavy metal (${R}_{q,P{b}^{2+}}$ = 0.95 and ${R}_{q,C{d}^{2+}}$ = 0.98) was affected at higher metal concentrations. This may be attributed to (1) the competitive adsorption between positively charged Pb^2+^ and Cd^2+^ ions and (2) steric hindrance effect between the heavy metals and the oxygen-containing groups such as –OH and –COOH/–COO− at high metal concentrations^56^.

Meanwhile, in the MV and Pb^2+^ multi-component system (Fig. 6B), the adsorption of both MV and Pb^2+^ were less affected at lower initial MV concentration (${R}_{q,P{b}^{2+}}$ and *R*_*q,MV*_ = 1). Basically, this indicates that the presence of MV does not pose a negative impact on the Pb^2+^ adsorption as there is no interaction between the Pb^2+^ ions and MV molecules when the initial concentration of MV was low (100 mg/L). However, at higher MV concentrations, even though MV removal is less affected (*R*_*q,MV*_ = 1), a slight decrease in Pb^2+^ adsorption (${R}_{q,P{b}^{2+}}$ = 0.95) was observed. This suggests that at higher concentrations, MV provides a stronger negative effect^55^. Consequently, Pb^2+^ ions are unable to access the available binding sites thereby leading to a slight reduction in the Pb^2+^ sorption capacity.

### Regeneration

To elucidate the practicability of the adsorbent, the reusability test of D-GSH was carried out using various solvents such as NaOH, HNO_3_ & acetone. The obtained results in Fig. 6C showed that an eluent solution of 0.1 M NaOH exhibited high efficiency in desorbing MV from spent D-GSH adsorbent. Even after 3 cycles, no significant reduction in adsorption capacity was observed. Acetone also desorbed MV dyes well, with a reduction in *q*_*e*_ value of <10% after 3^rd^ cycle. A similar trend was observed for the regeneration of CR-adsorbed D-GSH using both NaOH and acetone (Fig. 6D). On the other hand, for heavy metal adsorbed D-GH 0.1 M HNO_3_ seems to be the promising eluent with a reduction in *q*_*e*_ of <5% after the third cycle (Fig. 6E,F). Desorption using acetone also gave promising results as the reduction in the *q*_*e*_ value was less than 10%, indicating that acetone could be used as a solvent for regenerating both anionic as well as cationic pollutants adsorbed on D-GSH. Because of the remarkable capability as a dye and heavy metal scavenger along with its excellent regeneration ability, D-GSH outperforms all the other graphene sand hybrids so far reported in the literature (Table S4 in the Supplementary Information). Thus, it is the difference of structure, composition, morphology, and surface property of carbon precursor that has played a major role in improving the properties of the synthesized graphene sand hybrid material, introducing D-GSH prepared from date syrup as an efficient and reusable material for the removal of dyes and heavy metals from waters.

### Adsorptive interaction mechanism

**Figure 7:**
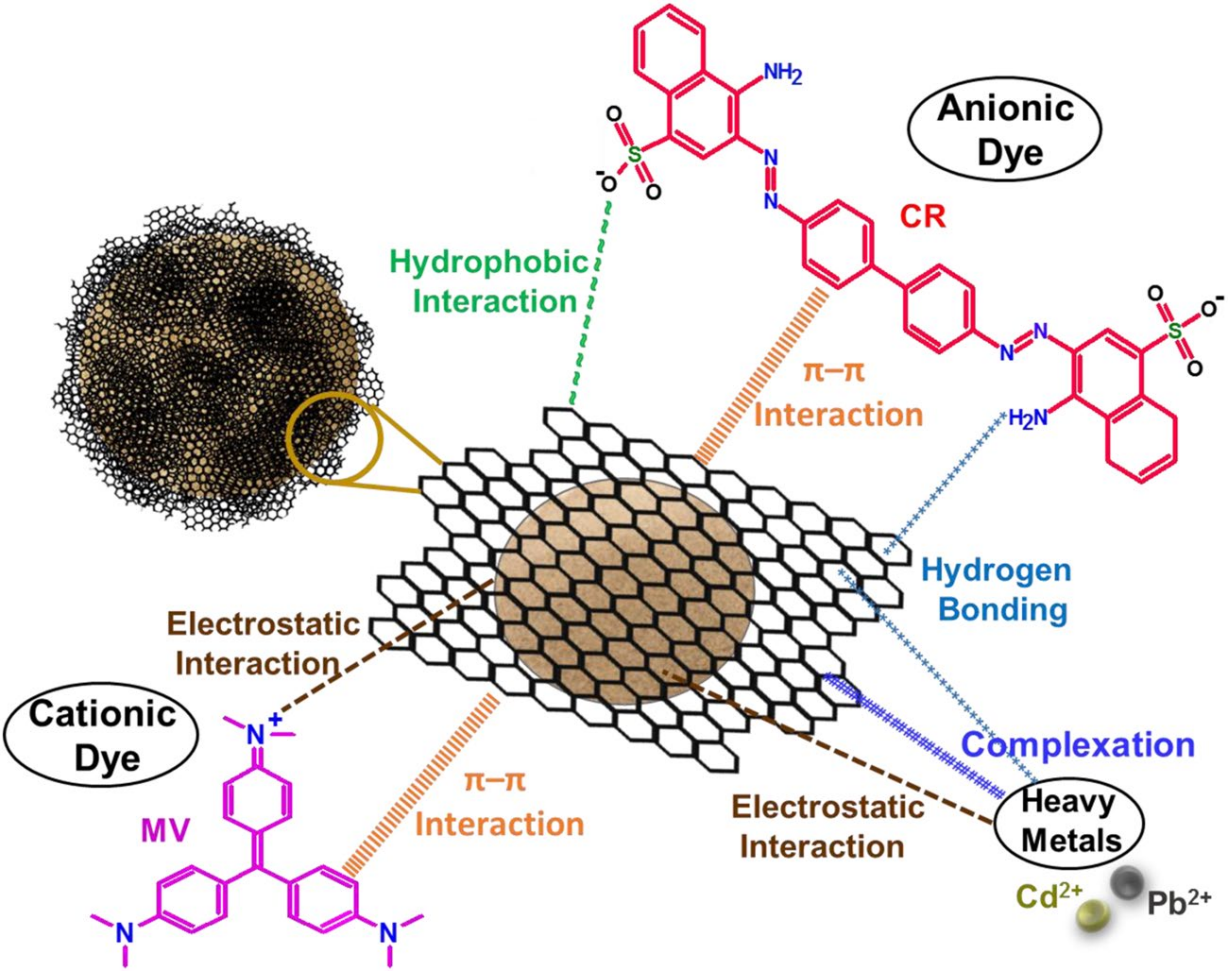
Plausible mechanism for the adsorption of cationic and anionic contaminants onto D-GSH.

To get a better perspective of the high extraction efficiency of D-GSH for both heavy metals and organic dyes, it is essential to unravel the interaction mechanisms involved in the adsorption process. Desert sand is a well-known inorganic substrate with a hydrophilic surface rich in -OH groups. After the immobilization of graphene on desert sand, the adsorption capacity of D-GSH has improved remarkably. The wrapping of graphene on sand is believed to open up the stacked interlamination of graphene and thereby exposing the powerful adsorption sites available in the interlayers. Furthermore, with the presence of a hydrophobic graphene sheet with sp^2^ hybridized structure on the sand surface, the hydrophilic sand surface becomes hydrophobic, negatively charged and possesses π-electron conjugated structure. Because of this highly negatively charged surface, the anionic D-GSH consequently showcased high extraction efficiencies for cationic dye pollutants such as MV rather than anionic dyes (CR). However, besides electrostatic interaction, the possibility of π-π bonding also played a crucial role in dye adsorption^57^. This was evident as the adsorption of anionic dyes was facilitated, even though the adsorbent and adsorbate had the same charge. This is attributed to the sp^2^ hybridized structure of graphene on D-GSH, which could present the possibility of π-π bonding with the aromatic rings of dyes contaminants. Additionally, van der Waals interactions such as hydrogen bonding between the carboxyl and hydroxyl groups on dyes and graphene as well as hydrophobic interactions are also believed to enhance the anionic dye adsorption capacity of D-GSH greatly^58^. Thus, the versatile chemistry of graphenes, such as the presence of negatively charged surface functional, abundant sp^2^ carbon sites and hydrophobic nature along with porous structure contributes to the extraordinary dye removal capacity showcased by D-GSH. Similar to dyes, heavy metal ion binding ability of D-GSH is primarily ascribed to its interconnected porous structure, which provides ample adsorption sites for heavy metal adsorption. The ion-chelating negatively charged surface functional groups present on D-GSH is believed to render the adsorption of heavy metals such as Pb^2+^ and Cd^2+^^53^^,^^54^. Moreover, the oxygen functional group present on the surface of D-GSH further enhances the metal adsorption via hydrogen bonding or complexation^59^. Thus, the wrapping of graphene on sand using date syrup as a precursor not only produced a versatile adsorbent with very high dye (anionic and cationic) and heavy metal adsorption capacity but also provided a facile green methodology to utilize graphene for water treatment. The whole plausible mechanism involved in the adsorption of cationic and anionic contaminants on to D-GSH composites is as shown in Fig. 7.

## Conclusion

A simple and facile *in-situ* strategy has been reported for the production of graphene sand hybrid using date syrup (D-GSH) via a one-step pyrolysis. The applicability of D-GSH as an eco-adsorbent for decontaminating wastewater was evaluated through batch adsorption using MV, CR and Pb2 +  and Cd2 +  as model pollutants. The so produced composite showcased highest adsorption capacity for MV (2564 mg/g), Pb^2+^ (781 mg/g) and Cd^2+^ (793 mg/g) at 25 °C along with a high adsorption capacity for CR (333 mg/g) and good recyclability (3 cycles). The appropriate adsorbent dosages for MV, CR, and heavy metals adsorption onto D-GSH were 5.0, 25.0 and 10.0 mg/10 mL, respectively at a shaking time of 45 min and temperature of 25 to 45 °C. Kinetically, the pseudo-second-order model well fitted the experimental data. Thermodynamically, the Langmuir model best described the equilibrium data for all the contaminants. The adsorption isotherm fitting and thermodynamic calculations suggest a strong electrostatic interaction between the cationic adsorbate (MV, Cd^2+^ and Pb^2+^) and the adsorbent. The highly hydrophobic effect and van der Waals interactions contributed to the adsorption of anionic contaminants such as CR. Furthermore, the D-GSH adsorbent showed remarkable efficiency in simultaneously removing both dye and heavy metals from the multi-component systems. Thus, the exceptional water-treatment capabilities of D-GSH confirm its great potential as an adsorbent material resource for water purification.

## Supplementary information

D-GSH Supplementary Information

## Data Availability

The authors declare that the data is available, and can be provided at any stage.
