# Supplementary material for: Sustainable synthesis of graphene-based adsorbent using date syrup
Source: Sci Rep. 2019 Dec 2;9:18106. doi: 10.1038/s41598-019-54597-x (PMC6889283; doi:10.1038/s41598-019-54597-x)
Supplement: Supplementary file 1 — D-GSH Supplementary Information [file 41598_2019_54597_MOESM1_ESM.pdf]

Supplementary information

Sustainable synthesis of graphene-based adsorbent using date syrup

Shaihroz Khan, Anjali Achazhiyath Edathil*, Fawzi Banat*,

Department of Chemical Engineering,

Khalifa University - SAN Campus, PO Box: 127788, Abu Dhabi, United Arab Emirates.

*Email of corresponding author: *fawzi.banat@ku.ac.ae*, *anjali.edathil@ku.ac.ae*

Materials and Methods:

Materials:

Date syrup was procured from local UAE market and desert sand particles were collected from the KU-SAN Campus, Abu Dhabi, UAE. Sand particles were sieved to the required particle size, cleaned using a magnet to remove the ferric impurities followed by thorough washing with deionized water (Milli Q plus, Merck Millipore Co., Germany) to remove any organic matter from plant and animals and finally dried overnight at 80 °C before use. Dyes such as methyl violet (MV, >98%), methylene blue (MB, > 96%), congo red (CR) and methyl orange (MO) were purchased from Acros Organics, USA. Heavy metal salts such as zinc acetate dihydrate ($Zn(C_2H_3O_2)_2{\cdot}2H_2O$, VWR Chemicals, 99% purity), copper sulphate pentahydrate ($CuSO_4$. $5H_2O$, Sigma Aldrich, 99% purity), lead nitrate ($Pb(NO_3)_2$, Merck, Germany) and cadmium chloride ($CdCl_2$, Sigma Aldrich, 99% purity) purchased were of analytical grade and used without any further modification. All other chemicals such as sodium hydroxide (NaOH), nitric acid (HNO_3) and acetone that were used for the regeneration of the adsorbent were analytical grade.

Structural Characterization

Raman spectra of D-GSH were characterized using a Jobin Yvon Horiba LabRAM spectrometer with back-scattered confocal configuration to monitor the transformation of date syrup to graphene-like nanosheet layers on desert sand particles. X-ray powder diffraction (XRD) was used to monitor the mineral phase of D-GSH and desert sand before graphene coating. The pattern spectrum was obtained using analytical X'Pert PRO Powder Diffractometer (Cu Kα radiation 1.5406 Å°, 40 kV, 40 mA) at a diffraction angle (2θ) ranging from 5°to 80° and a step size of 0.02°. The surface area and pore size distribution of D-GSH were analyzed by the Brunauer–Emmett–Teller (BET) method using N_2 gas on an Autosorb-1, Quantachrome surface analyzer at 77K. D-GSH samples were degassed under vacuum for 15 h at 453 K prior to the analysis. In brief, the surface area was determined by the Brunauer–Emmett–Teller (BET) method between the relative pressures of 0.0 to 1.0 using the nitrogen adsorption-desorption isotherms obtained based on the monolayer adsorption theory, while the pore distribution was calculated by fitting the isotherms according to the Barrett–Joyner–Halenda (BJH) method. Fourier Transform-Infrared Spectroscopy was performed using KBr technique (Bruker Vertex 70) in the 500-4000 cm^{-1} region with a resolution of 1 cm^{-1} to investigate the changes in the functional groups of D-GSH before, after adsorption and regeneration. The surface morphological study and energy dispersive X-ray spectroscopy (EDX) analysis of D-GSH was carried out using a scanning electron microscope equipped with EDX analyzer (SEM-EDX, FEG Quanta-250). After adhering the samples on the stubs which have double-coated carbon-conductive tabs, the stub was gold-platinum sputter coated to enhance the sample conductivity prior to SEM analysis. The microstructure of the D-GSH was evaluated using Transmission electron microscopy (TEM) images using an FEI Tecnai 20

operating at 200 kV. To understand the distribution of graphene over sand, D-GSH sample was sonicated in ethanol, and few drops of supernatant solution was transferred to 400 mesh formvar copper electron microscopy grids (coated with holey carbon film) and the TEM images was captured using Digital Micrograph software (Gatan, USA).

Effect of experimental conditions on adsorption

To access the effect of various experimental conditions on adsorption, batch experiments were performed by varying sand particle size, adsorbent dosage, solution volume, pH, time, initial concentration and temperature. The effect of varying sand particle size were studied by suspending 25.0 mg of D-GSH composites prepared using desert sand of particle size such as 0 to 200, 200 to 500, 500-1000 and 1000 to 1500 μm in solution having initial MV or CR dye concentration of 200 mg/L and equilibrating for 240 min at 25 ± 0.5 °C. At the same initial dye concentration and conditions, the effect of varying amounts of D-GH (5.0 to 30 mg) and solution volume (10.0 and 20.0 mL at two different dosage of 5.0 and 25.0 mg) on the adsorption were studied. Whereas in case of heavy metals, the effect of adsorbent dosage (5.0 to 25.0 mg) was studied at two different initial concentration of 10.0 and 100.0 mg/L. To understand the effect of pH, 25.0 mg of D-GH was added to 200 mg/L heavy metal solution whose pH were varied from 2 to 12 by adjusting with 0.1 M HCl and NaOH before adding the adsorbent.

For studying the effect of contact time on MV and CR dye adsorption, sorption kinetic experiments were conducted at initial aqueous dye concentration of 200.0 and 1000.0 mg/L for MV and 200.0 and 500.0 mg/L for CR and supernatants were sampled at different sorption duration. Firstly, 5.0 and 25.0 mg of D-GSH was transferred into each of the 25 mL conical flasks containing 10.0 mL of MV and CR dye solution, respectively. On the other hand, the effect of contact time on Pb^{2+} and Cd^{2+} heavy metals adsorption was studied by suspending 10.0 mg of D-GSH in 10.0 mL of heavy metal aqueous solution having initial concentration of 10.0 mg/L. The system were then shaken at 140 rpm at room temperature (25.0 ± 0.5 ºC) on a water bath for contact time of 1, 3, 5, 10, 15, 30, 45, 60, 120 and 240 min. After the predetermined period of sorption time had elapsed, supernatant was collected and analyzed for residual dye concentration. To obtain the adsorption isotherms, a known weight of D-GSH adsorbent was severally added into a set of 10.0 mL solutions with a series concentration of adsorbates and equilibrated for the required contact time at 25.0, 35.0 and 45.0 ºC, respectively. All the adsorption experiments were carried out in duplicates and the average of the results are reported.

Regeneration and Reusability Test

After dye and heavy metal adsorption experiments, regeneration was performed by rinsing and washing the contaminant-loaded D-GH with various eluents such as 1.0 M NaOH and acetone for dye loaded D-GH or

0.1 M HNO_3 and acetone for heavy metal loaded D-GH, followed by washing with deionized water. Then, the regenerated and dried D-GSH was reused for further adsorption experiments. This adsorption-desorption cycle was repeated for three cycles.

Selectivity test:

To access the selectivity of D-GSH for different metal ions, simultaneous adsorption experiments were designed with multi-component systems consisting of Cd^{2+} as competing ion and Pb^{2+} as target ion. 10.0 mg of D-GSH was added to 10.0 ml of each solution containing 10 and 100 mg/L of Pb^{2+} and Cd^{2+} ions and equilibrated by shaking on a temperature regulated water bath (Dihan, Korea) at a constant speed of 140 rpm at 25 ˚C. The concentration of each metal ion was analyzed using ICP-OES and the adsorption capacity was calculated. Similarly, to identify the selectivity of D-GH for organic dye over heavy metals, 10.0 mg of D-GH was added to 10.0 ml solution each containing 100 and 1000 mg/L of Pb^{2+} ion and MV dye.

Data analysis of sorption kinetics and isotherm

To deeply characterize the inherent physio-chemical process involved in the adsorption of dye and heavy metal contaminant onto D-GSH and to elucidate the adsorptive performance of D-GSH at varying initial concentration and temperature, the experimental adsorption kinetics and equilibrium isotherms, respectively were analyzed using various kinetic and isotherm models. Furthermore, to determine the practical applicability of the adsorption process, the obtained adsorption isotherms were utilized to predict the thermodynamic parameters. The details of the working formula for various kinetic and isotherm models including the equations and model parameters along with the equations for performing thermodynamic analysis are given below:

Pseudo-first-order model[1]

$$\ln(q_e - q_t) = ln q_e - k_1 t$$

Where, q_t is the adsorption capacities of D-GSH at any time 't' (mg/g), adsorption capacities of D-GCS at equilibrium (mg/g), $k_1$is the pseudo-first-order rate constant (min^{-1}) and t is the contact time.

Pseudo-second-order model[2]

$$\frac{t}{q_t} = \frac{1}{k_2 * q_e^2} + \frac{t}{q_e}$$

Where, $k_2$is the pseudo-second-order rate constant (g mg^{-1} min^{-1})

Langmuir isotherm[3]

$$\frac{1}{q_e} = \frac{1}{q_{max}} + \frac{1}{q_{max} K_L C_e}$$

Where, q_e is the dye/heavy metal equilibrium adsorption ability (mg/g), C_e is the equilibrium concentration of dye/heavy metal in the solution (mg/L) and q_{max} is the maximum dye/heavy metal adsorption capacity (mg/g).

Freundlich Isotherm[4]

$$log q_e - log K_f = \frac{1}{n} log C_e$$

Where, K_f is the Freundlich constant ($mg^{1-1/n} L^{1/n} g^{-1}$) and $\frac{1}{n}$ is the heterogeneity parameter, an estimate of the intensity of adsorption.

Tempkin Isotherm[5]

$$q_e = \frac{RT}{b_T} ln\, A_T\, C_e$$

Where, R is the Universal gas constant (8.314 J K^{-1} mol^{-1}), T is the Absolute temperature (K), b_T is the Temkin isotherm constant related to heat of adsorption (J mol^{-1}) and A_T is the Temkin isotherm equilibrium binding constant (L g^{-1}).

Thermodynamic Study

$$\Delta G = -RT ln K_L$$

$$ln K_L = \frac{\Delta S}{R} - \frac{\Delta H}{RT}$$

Where, ΔG is the Gibb's free energy change (kJ mol^{-1}), ΔS is the standard entropy change (J mol^{-1}) and ΔH is the standard enthalpy change (kJ mol^{-1}).

Figures

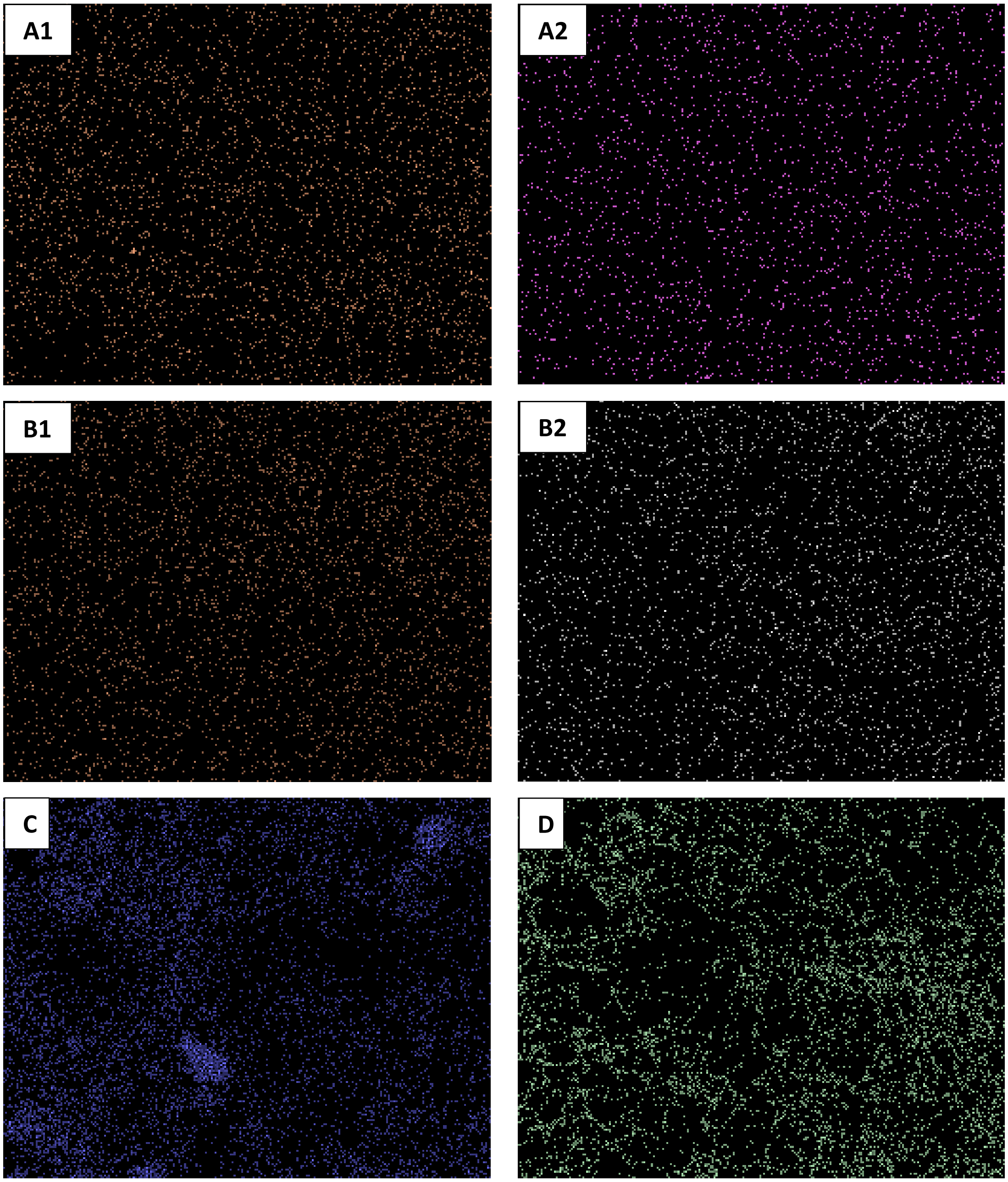

Figure S1 Elemental mapping of (A) MV, (B) CR (C) Pb and (D) Cd adsorbed D-GH samples to study the uniform distribution of adsorbed species throughout the hybrid sand surface (nitrogen colored in orange, chlorine colored in pink, sulfur colored in white, lead colored in violet and cadmium colored in pista green).

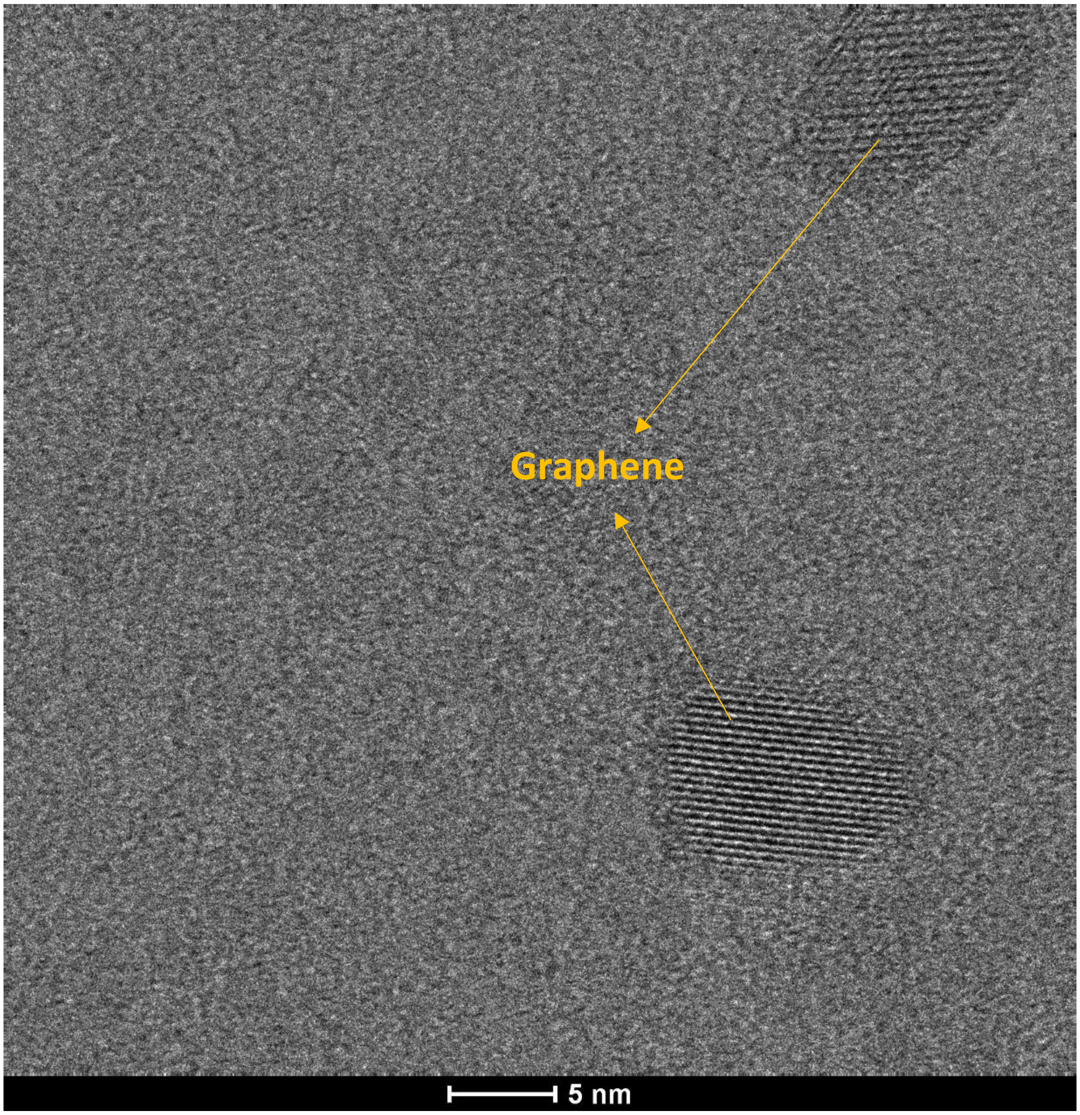

Figure S2 HR-TEM image of D-GSH.

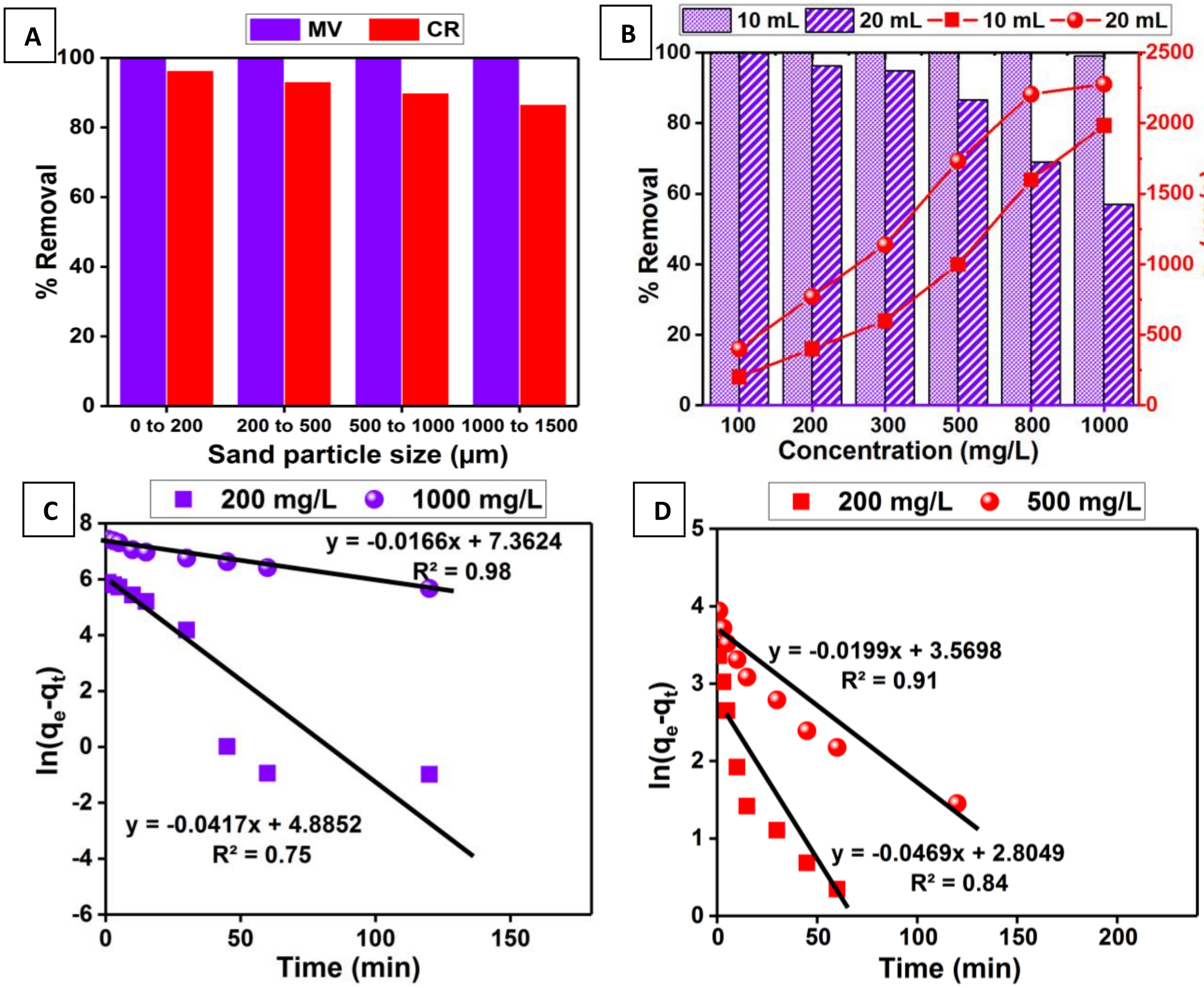

Figure S3 Dye adsorption results: (A) Effect of sand particle size (B) Effect of solution volume on MV adsorption, (C) and (D) Plots of pseudo-first order kinetic models for MV and CR, respectively. [Straight lines are the fitting results].

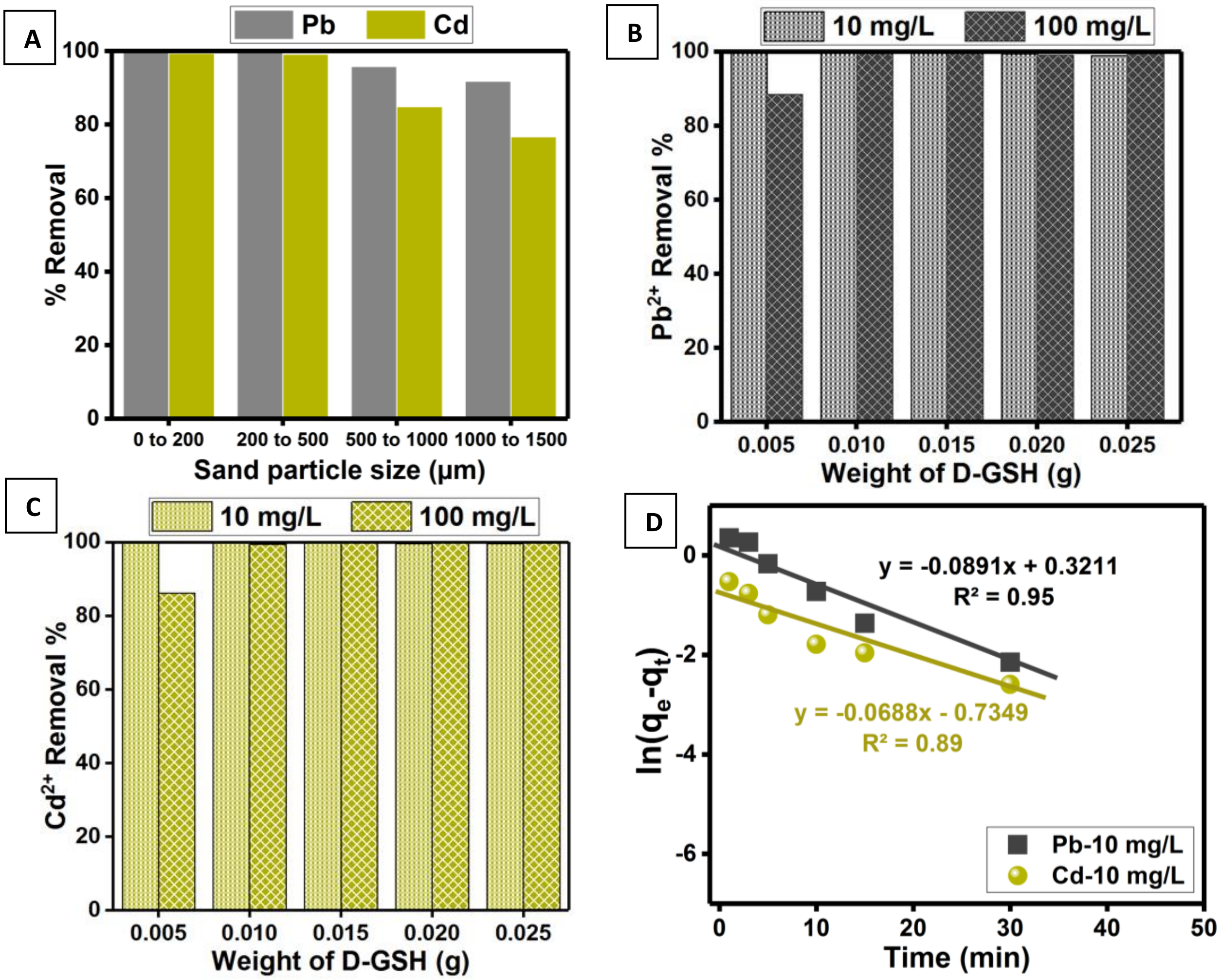

Figure S4 Heavy metal adsorption results: (A) Effect of sand particle size, (B) and (C) Effect of adsorbent dosage on Pb^{2+} and Cd^{2+} adsorption, respectively, (D) Plots of pseudo-first order kinetic models [Straight lines are the fitting results].

Tables:

Table S1 Fitted parameters obtained from various kinetic models for MV and CR adsorption onto D-GH and its comparison with experimentally obtained data.

**Type of Model**	**Parameters**	**MV**		**CR**		**Pb**	**Cd**
	**C_0 (mg/L)**	**200**	**1000**	**200**	**500**	**10**	**10**
**Experimental**	$q_{e(exp)}$(mg g^{-1})	399.16	1987.98	79.67	199.56	9.94	9.97
**Pseudo-First-Order Model**[1] $[\ln(q_e - q_t) = ln q_e - k_1 t]$	$q_{e(Cal)}$(mg g^{-1})	401.78	1989.82	16.53	35.51	1.38	2.09
	Rate constant, k_1 (min^{-1})	0.06	0.02	0.05	0.02	0.09	0.07
	R^2	**0.99**	**0.99**	0.84	0.91	0.95	0.89
**Pseudo-Second-Order Model**[2] $\left[\frac{t}{q_t} = \frac{1}{k_2 * {q_e}^2} + \frac{t}{q_e}\right]$	$q_{e(Cal)}$(mg g^{-1})	555.56	1666.67	80.00	200.00	9.96	9.87
	Rate constant, k_2 (g mg^{-1} min^{-1})	8.78E-05	5.00E-05	1.12E-02	2.75E-03	2.90E-01	7.08E-01
	R^2	0.95	0.98	**1.00**	**0.99**	**1.00**	**1.00**

Table S2 Comparison with other graphene based adsorbents for the removal of MV and CR dyes.

**Graphene based adsorbents**	**Dye**	**q_e (mg g^{-1})**	**Ref.**
Co_3O_4/graphene nanocomposite (CoOIG)	MV	34.48	[6]
Nano-graphite/Fe_3O_4 composite (NG-FC)		144.72	[7]
Graphene oxide magnetic composite gel (mGO/PVA CGs)		221.23	[8]
Three dimensional graphene oxide nanostructure (3D-GS)		467	[9]
Magnetic Fe_3O_4 graphene nanocomposite (FGC)	CR	33.66	[10]
Magnetic mesoporous titanium dioxide–graphene oxide (Fe_3O_4@mTiO_2@GO)		89.95	[11]
Graphene oxide-chitosan fibers (GO-CS)		294.12	[12]
Date syrup based graphene sand hybrid (D-GSH)	MV<br>CR	2564.10<br>333.33	**This work**

Table S3 Comparison with other graphene based adsorbents for the removal of Cd^{2+} and Pb^{2+} heavy metals

**Graphene based adsorbents**	**Metal**	**q_e (mg g^{-1})**	**Ref.**
Graphene oxide (GO)	Cd^{2+}	23.9	[13]
Magnetic graphene oxide (MGO)		91.29	[14]
Few layered graphene oxide (FLGO)		106.3	[15]
Graphene oxide (GO)	Pb^{2+}	125	[16]
Exfoliated graphene nanosheet (GNS)		22.42	[17]
SiO_2/Graphene composite		113.6	[18]
Mesoporous silica (SBA-15)-grafted graphene oxide (GO–SBA-15)		255.10	[19]
Magnetic cobalt ferrite-reduced graphene oxide nanocomposite ($CoFe_2O_4$/RGO)		299.4	[20]
Graphene/R-FeOOH		373.8	[21]
Date syrup based graphene sand hybrid (D-GSH)	Cd^{2+}<br>Pb^{2+}	793.65<br>781.25	**This work**

Table S4 Comparison with other graphene sand hybrids

**Graphene Sand hybrids**	**Type of carbon source**	**Contaminants**		**q_e (mg g^{-1})**	**Ref.**
		**Dyes**	**Heavy Metals**		
GO_{SAND}	Graphite		Hg^{2+}	236.96	[22]
MGSC	Arenga palm sugar	-	Ni^{2+}	6.36	[23]
NCCS	Table sugar	MV	-	49.03	[24]
GSC	Sugar	R6G	-	55	[25]
GSC	Asphalt	R6G	-	75.4	[26]
D-GSH	Date Syrup	MV<br>CR	 <br>Pb^{2+}<br>Cd^{2+}	2564.10<br>333.33<br>781.25<br>793.65	**This work**

References:

1 Lagergren, S. About the theory of so-called adsorption of soluble substances. (1898).

2 Ho, Y.-S. & McKay, G. Pseudo-second order model for sorption processes. *Process biochemistry* **34**, 451-465 (1999).

3 Langmuir, I. The adsorption of gases on plane surfaces of glass, mica and platinum. *Journal of the American Chemical society* **40**, 1361-1403 (1918).

4 Freundlich, H. Over the adsorption in solution. *J. Phys. Chem* **57**, 1100-1107 (1906).

5 Temkin, M. & Pyzhev, V. Kinetics of ammonia synthesis on promoted iron catalysts. *Acta physiochim. URSS* **12**, 217-222 (1940).

6 Muralisankar, I., Agilan, S., Selvakumar, R. & Vairam, S. Synthesis of Co3O4/graphene nanocomposite using paraffin wax for adsorption of methyl violet in water. *IET nanobiotechnology* **12**, 787-794 (2018).

7 Li, C., Dong, Y., Yang, J., Li, Y. & Huang, C. Modified nano-graphite/Fe3O4 composite as efficient adsorbent for the removal of methyl violet from aqueous solution. *Journal of Molecular Liquids* **196**, 348-356 (2014).

8 Cheng, Z. *et al.* One-step fabrication of graphene oxide enhanced magnetic composite gel for highly efficient dye adsorption and catalysis. *ACS Sustainable Chemistry & Engineering* **3**, 1677-1685 (2015).

9 Liu, F., Chung, S., Oh, G. & Seo, T. S. Three-dimensional graphene oxide nanostructure for fast and efficient water-soluble dye removal. *ACS applied materials & interfaces* **4**, 922-927 (2012).

10 Yao, Y. *et al.* Synthesis, characterization, and adsorption properties of magnetic Fe3O4@ graphene nanocomposite. *Chemical Engineering Journal* **184**, 326-332 (2012).

11 Li, L., Li, X., Duan, H., Wang, X. & Luo, C. Removal of Congo Red by magnetic mesoporous titanium dioxide–graphene oxide core–shell microspheres for water purification. *Dalton Transactions* **43**, 8431-8438 (2014).

12 Du, Q. *et al.* Highly enhanced adsorption of congo red onto graphene oxide/chitosan fibers by wet-chemical etching off silica nanoparticles. *Chemical Engineering Journal* **245**, 99-106 (2014).

13 Bian, Y. *et al.* Effect of the oxygen-containing functional group of graphene oxide on the aqueous cadmium ions removal. *Applied Surface Science* **329**, 269-275 (2015).

14 Deng, J.-H. *et al.* Simultaneous removal of Cd (II) and ionic dyes from aqueous solution using magnetic graphene oxide nanocomposite as an adsorbent. *Chemical Engineering Journal* **226**, 189-200 (2013).

15 Zhao, G., Li, J., Ren, X., Chen, C. & Wang, X. Few-layered graphene oxide nanosheets as superior sorbents for heavy metal ion pollution management. *Environmental science & technology* **45**, 10454-10462 (2011).

16 Huang, X. & Pan, M. The highly efficient adsorption of Pb (II) on graphene oxides: A process combined by batch experiments and modeling techniques. *Journal of molecular liquids* **215**, 410-416 (2016).

17 Huang, Z.-H. *et al.* Adsorption of lead (II) ions from aqueous solution on low-temperature exfoliated graphene nanosheets. *Langmuir* **27**, 7558-7562 (2011).

18 Hao, L. *et al.* SiO2/graphene composite for highly selective adsorption of Pb (II) ion. *Journal of colloid and interface science* **369**, 381-387 (2012).

19 Li, X., Wang, Z., Li, Q., Ma, J. & Zhu, M. Preparation, characterization, and application of mesoporous silica-grafted graphene oxide for highly selective lead adsorption. *Chemical Engineering Journal* **273**, 630-637 (2015).

20 Zhang, Y. *et al.* Adsorption of Pb (II) and Hg (II) from aqueous solution using magnetic CoFe2O4-reduced graphene oxide. *Journal of Molecular Liquids* **191**, 177-182 (2014).

21 Cong, H.-P., Ren, X.-C., Wang, P. & Yu, S.-H. Macroscopic multifunctional graphene-based hydrogels and aerogels by a metal ion induced self-assembly process. *ACS nano* **6**, 2693-2703 (2012).

22 Gao, W. *et al.* Engineered graphite oxide materials for application in water purification. *ACS applied materials & interfaces* **3**, 1821-1826 (2011).

23 Zularisam, A. & Wahida, N. in *IOP Conference Series: Materials Science and Engineering.* 012006 (IOP Publishing).

24 Moradi, S. & Azizian, S. Preparation of nanostructured carbon covered sand for removal of methyl violet from water. *Journal of Molecular Liquids* **219**, 909-913 (2016).

25 Gupta, S. S., Sreeprasad, T. S., Maliyekkal, S. M., Das, S. K. & Pradeep, T. Graphene from sugar and its application in water purification. *ACS applied materials & interfaces* **4**, 4156-4163 (2012).

26 Sreeprasad, T. S., Gupta, S. S., Maliyekkal, S. M. & Pradeep, T. Immobilized graphene-based composite from asphalt: Facile synthesis and application in water purification. *Journal of hazardous materials* **246**, 213-220 (2013).
